# Slow left atrial conduction velocity in the anterior wall calculated by electroanatomic mapping predicts atrial fibrillation recurrence after catheter ablation—Systematic review and meta‐analysis

**DOI:** 10.1002/joa3.13146

**Published:** 2024-09-05

**Authors:** Antonia Anna Lukito, Wilson Matthew Raffaello, Raymond Pranata

**Affiliations:** ^1^ Department of Cardiology and Vascular Medicine, Siloam Hospitals Lippo Village—Faculty of Medicine Universitas Pelita Harapan Tangerang Indonesia

**Keywords:** atrial fibrillation, catheter ablation, conduction velocity, electroanatomic mapping, recurrence

## Abstract

**Background:**

This study aimed to investigate and perform diagnostic test meta‐analysis on whether slow left atrial conduction velocity (LACV) in the anterior wall calculated by electroanatomic mapping predicts atrial fibrillation (AF) recurrence after catheter ablation.

**Methods:**

Extensive literature search was performed on PubMed, SCOPUS, and EuropePMC up to June 5, 2024. The exposure group included AF patients with slow LACV in the anterior wall, while the control group included AF patients without slow LACV in the anterior wall. Slow LACV in the anterior wall was defined as LACV below study‐specific cut‐off points in m/s, measured by invasive electroanatomic mapping. The primary outcome of this study was AF recurrence, defined as AF/Atrial Flutter/Atrial Tachyarrhythmias lasting over 30 s at least 3 months after the blanking period postablation.

**Results:**

This systematic review and meta‐analysis included seven studies, involving a sample size of 1428 patients with mean follow‐up duration were 13 months. Patients with AF recurrence has slower LACV in the anterior wall (mean difference − 0.16 m/s [−0.18, −0.15], *p* < .001). Slow LACV in the anterior wall defined as LACV below 0.70–0.88 m/s was associated with increased AF (adjusted OR 3.41 [1.55, 7.50], *p* = .002). Slow LACV in the anterior wall has an AUROC of 0.80 [0.76–0.83], sensitivity of 70% [52, 84], specificity of 76% [67, 83], positive likelihood ratio of 2.9 [2.3, 3.6], negative likelihood ratio of 0.39 [0.25, 0.63] for predicting AF recurrence postablation.

**Conclusion:**

Slow LACV in the anterior wall was associated with AF recurrence after catheter ablation.

## Graphical abstract

Slow LACV in the anterior wall defined as LACV below 0.70–0.88 m/s was associated with more than threefold increased AF recurrence, has an AUROC of 0.80, sensitivity of 70% and specificity of 76%. This finding can identify potentially relevant re‐entry circuits, which may serve as ablation targets.

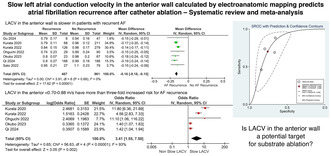

## 1 INTRODUCTION

Atrial fibrillation (AF) is one of the most frequently encountered cardiac arrhythmias in clinical practice. It is associated with high mortality and morbidity and imposes an economic burden due to increasing hospitalization costs. AF is a progressive condition that is challenging to treat. Advances in AF ablation technology have produced acceptable results, particularly for paroxysmal AF.^1^, ^2^, ^3^, ^4^ However, AF recurrence remains a significant concern, especially in persistent AF. Additional ablation strategies beyond pulmonary vein isolation (PVI) are being explored, but with limited success. Therefore, the search for new strategies continues to be a priority.

P‐wave duration, which represents interatrial conduction, has been shown to predict AF recurrence after ablation.^5^ Investigating left atrial conduction velocity (LACV) and its pathways may enable the localization of the most critical regions. The LACV of the anterior wall shows promising results for predicting AF recurrence.^6^, ^7^, ^8^, ^9^, ^10^, ^11^, ^12^ Identifying these key areas might help with risk stratification and the development of relevant ablation strategies. Existing studies show varying model performance; therefore, it is important to perform a pooled analysis of these studies. This study aims to investigate and conduct a diagnostic test meta‐analysis to determine whether slow LACV in the anterior wall, as calculated by electroanatomic mapping, predicts AF recurrence after catheter ablation.

## 2 METHODS

### 2.1 Literature search strategy

Two reviewers independently conducted an extensive literature search using the specified keywords: ((slow left atrial conduction velocity) OR (low left atrial conduction velocity) OR (long left atrial conduction velocity)) AND (atrial fibrillation) AND (ablation). The search was performed on PubMed, SCOPUS, and EuropePMC up to June 5, 2024. Any discrepancies were resolved through discussions. The eligibility of the records was assessed using predetermined inclusion and exclusion criteria.

**FIGURE 1:**
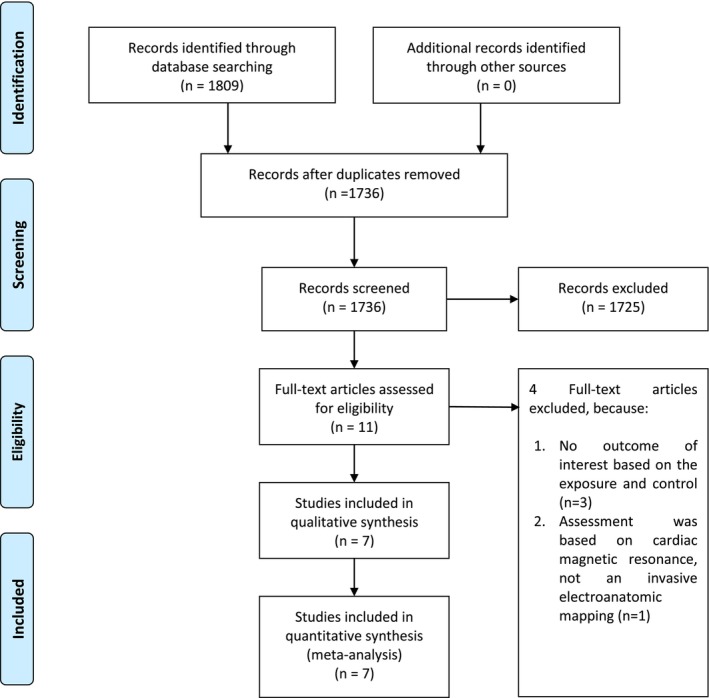
PRISMA Flowchart.

The authors conducted a systematic review in accordance with the Preferred Reporting Items for Systematic Reviews and Meta‐Analyses (PRISMA) guidelines. The protocol is registered in PROSPERO CRD42024556434. Figure 1 presents a flowchart illustrating the literature search process.

### 2.2 Exposure and control groups

The exposure group included AF patients with slow LACV in the anterior wall, while the control group included AF patients without slow LACV in the anterior wall. Slow LACV in the anterior wall was defined as LACV below study‐specific cut‐off points in m/s, measured by invasive electroanatomic mapping. The wavefront propagation in the anterior wall started from the Bachmann bundle, passed through the anterior wall toward the atrial appendage, crossed the left atrial appendage's ostium, and finally reached the isthmus of the mitral valve.^1^ Studies involving only PVI as well as those performing PVI with additional ablation beyond PVI were considered.

### 2.3 Selection criteria

We included observational studies (both prospective and retrospective) that reported the incidence of AF recurrence, comparing patients with slow LACV in the anterior wall to those without. We excluded abstract only articles, animal studies, letters to the editor, review articles, case reports, and non‐English language articles.

### 2.4 Outcome

The primary outcome of this study was AF recurrence, defined as AF/atrial flutter/atrial tachyarrhythmias lasting over 30 s at least 3 months after the blanking period postablation. A continuous variable analysis (mean difference) was performed for LACV in the anterior wall in patients with AF recurrence compared to those without. Adjusted hazard ratios (HR) and adjusted odds ratios (OR) were converted into adjusted ORs for comparison between patients with slow LACV in the anterior wall and those without.

### 2.5 Data extraction

Two reviewers independently extracted data using a form detailing the primary outcome, study design, sample size, inclusion criteria, percentage of paroxysmal AF, additional ablation beyond PVI, follow‐up duration, and age. The risk of bias in the studies was independently assessed by two reviewers using the Newcastle‐Ottawa Scale for observational studies. Any disagreements were resolved through discussion.

### 2.6 Statistical analysis

This meta‐analysis was performed using ReviewManager 5.4.1 and STATA 17. A random‐effects model approach using the inverse variance method was used for both mean difference and adjusted OR. Statistically significant heterogeneity was defined as an *I* ^2^ value >50% and/or a *p*_heterogeneity_ below .10. The pooled effect was considered statistically significant when the *p*‐value was less than .05. Egger's test was used to quantitatively measure small‐study effects. A diagnostic test meta‐analysis was performed to calculate the area under the receiver operating characteristic (AUROC), sensitivity, specificity, positive/negative likelihood ratios, and diagnostic OR. Funnel‐plot analysis qualitatively assessed small‐study effects and publication bias. A leave‐one‐out sensitivity analysis was performed to test statistical robustness.

## 3 RESULTS

This systematic review and meta‐analysis included seven studies, involving a sample size of 1428 patients (Figure 1).^6^, ^7^, ^8^, ^9^, ^10^, ^11^, ^12^ Among these studies, there are four prospective and three retrospective observational studies. The baseline characteristics of the included studies are detailed in Table 1. Two out of six studies performed additional ablation while the other four studies did not. By comparing studies with additional ablation beyond PVI and those who do not, additional ablation does not lower the rate of AF recurrence. Kurata (2022) reported that among patients with low‐voltage areas, 34% underwent low‐voltage area ablation, 5% underwent anterior line ablation, 8% underwent roof line ablation, and 3% underwent bottom line ablation. In Ohguchi (2022), additional ablation did not reduce the number of clinical recurrences. However, there was no indication that the additional ablation strategy was devised based on slow LACV in the anterior wall. Mean follow‐up duration was 13 months.

**TABLE 1:** Baseline characteristics of the included studies.

Author	Design	Sample size	Inclusion criteria	Cut‐off (m/s)	Age (years)	Paroxysmal (%)	Additional ablation	LACV assessment	LA diameter (mm)	Recurrence postablation	Mean FU (months)	NOS
Gu 2024	Unclear, possibly RC	53	Initial ablation, PVI only, Radiofrequency	None for anterior	62	62	No	No pacing	38	11%	8	7
Kurata 2020	Unclear, possibly PC	279	Initial ablation, PVI only, Radiofrequency/Cryoballoon/Laser	0.87	69	53	No	Paced rhythm from the high right atrium	39	24%	14	8
Kurata 2022	Unclear, possibly PC	405	Initial ablation, Radiofrequency/Cryoballoon/Laser	0.83	68	63	Yes	Paced rhythm from the high right atrium	41	31%	16	8
Ohguchi 2022	RC	119	Initial ablation, Radiofrequency	0.8	68	48	Yes	No pacing	40	24%	12	8
Okubo 2023	PC	90	Initial ablation, PVI only, Radiofrequency	0.7	65	46	No	Paced rhythm from the high right atrium	41	25%	17	8
Qi 2024	PC	188	Initial ablation, PVI only, Radiofrequency	0.887	67	64	No	No pacing	40	36%	13	8
Sato 2022^a^	RC	106	Initial ablation, PVI only, Radiofrequency	1.08	66	54	No	No pacing	40	25%	12	7

^a^ No adjusted odds/hazard ratio for LACV in the anterior wall.

### 3.1 Atrial fibrillation recurrence

**FIGURE 2:**
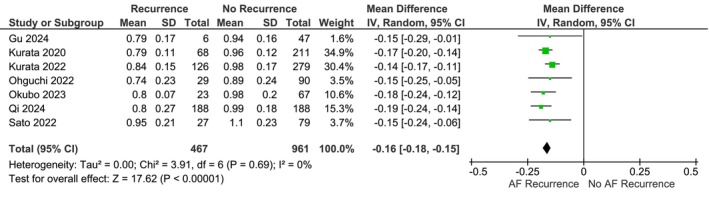
LACV in the anterior wall of patients with AF recurrence versus those without [continuous variable]. LACV, left atrial conduction velocity; AF, atrial fibrillation.

**FIGURE 3:**
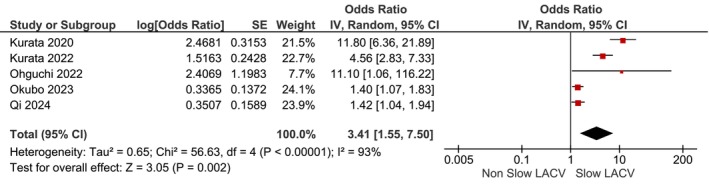
Slow LACV in the anterior wall and AF recurrence [adjusted odds ratio]. LACV, left atrial conduction velocity; AF, atrial fibrillation.

**FIGURE 4:**
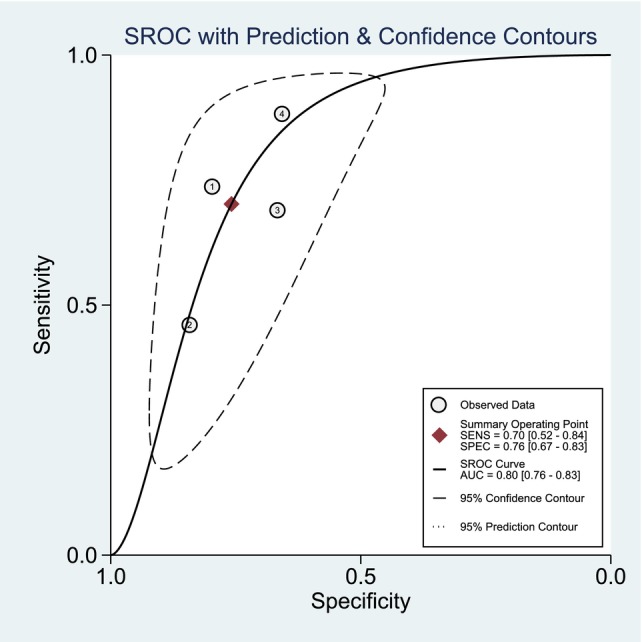
Area under the receiver operating characteristic (AUROC), sensitivity, and specificity for slow LACV in the anterior wall and AF recurrence. AF, atrial fibrillation; LACV, left atrial conduction velocity.

Patients with AF recurrence has slower LACV in the anterior wall (mean difference − 0.16 m/s [−0.18, −0.15], *p* < .001; *I* ^2^: 0%, *p*_heterogeneity_ = .69) (Figure 2). Slow LACV in the anterior wall defined as LACV below 0.70–0.88 m/s was associated with increased AF (adjusted OR 3.41 [1.55, 7.50], *p* = .002; *I* ^2^: 93%, *p*_heterogeneity_ < .001) (Figure 3). Leave‐one‐out sensitivity analysis showed that patients with AF recurrence has slower LACV in the anterior wall for both mean difference and adjusted OR (Table 2). Slow LACV in the anterior wall has an AUROC of 0.80 [0.76–0.83], sensitivity of 70% [52, 84], specificity of 76% [67, 83], Positive Likelihood Ratio of 2.9 [2.3, 3.6], Negative Likelihood Ratio of 0.39 [0.25, 0.63], Diagnostic OR of 7 [4, 13] for predicting AF recurrence postablation (Figure 4).

**TABLE 2:** Leave‐one‐out sensitivity analysis.

Study left out	Effect estimates
Mean Difference (m/s)	
Gu 2024	mean difference − 0.16 m/s [−0.18, −0.15], *p* < .001; *i* ^2^: 0%, *p*_heterogeneity_ = .57
Kurata 2020	mean difference − 0.16 m/s [−0.18, −0.14], *p* < .001; *i* ^2^: 0%, *p*_heterogeneity_ = .61
Kurata 2022	mean difference − 0.17 m/s [−0.20, −0.15], *p* < .001; *i* ^2^: 0%, *p*_heterogeneity_ = .95
Ohguchi 2022	mean difference − 0.16 m/s [−0.18, −0.15], *p* < .001; *i* ^2^: 0%, *p*_heterogeneity_ = .57
Okubo 2023	mean difference − 0.16 m/s [−0.18, −0.14], *p* < .001; *i* ^2^: 0%, *p*_heterogeneity_ = .62
Qi 2024	mean difference − 0.16 m/s [−0.18, −0.14], *p* < .001; *i* ^2^: 0%, *p*_heterogeneity_ = .79
Sato 2022	mean difference − 0.16 m/s [−0.18, −0.15], *p* < .001; *i* ^2^: 0%, *p*_heterogeneity_ = .57
Adjusted OR	
Kurata 2020	adjusted OR 2.23 [1.20, 4.14], *p* = .01; *I* ^2^: 86%, *p*_heterogeneity_ < .001
Kurata 2022	adjusted OR 3.15 [1.27, 7.81], *p* = .01; *I* ^2^: 93%, *p*_heterogeneity_ < .001
Ohguchi 2022	adjusted OR 3.09 [1.36, 6.99], *p* = .007; *I* ^2^: 95%, *p*_heterogeneity_ < .001
Okubo 2023	adjusted OR 4.72 [1.53, 14.54], *p* = .007; *I* ^2^: 93%, *p*_heterogeneity_ < .001
Qi 2024	adjusted OR 4.71 [1.49, 14.85], *p* = .008; *I* ^2^: 94%, *p*_heterogeneity_ < .001

Abbreviation: OR, odds ratio.

### 3.2 Publication bias

**FIGURE 5:**
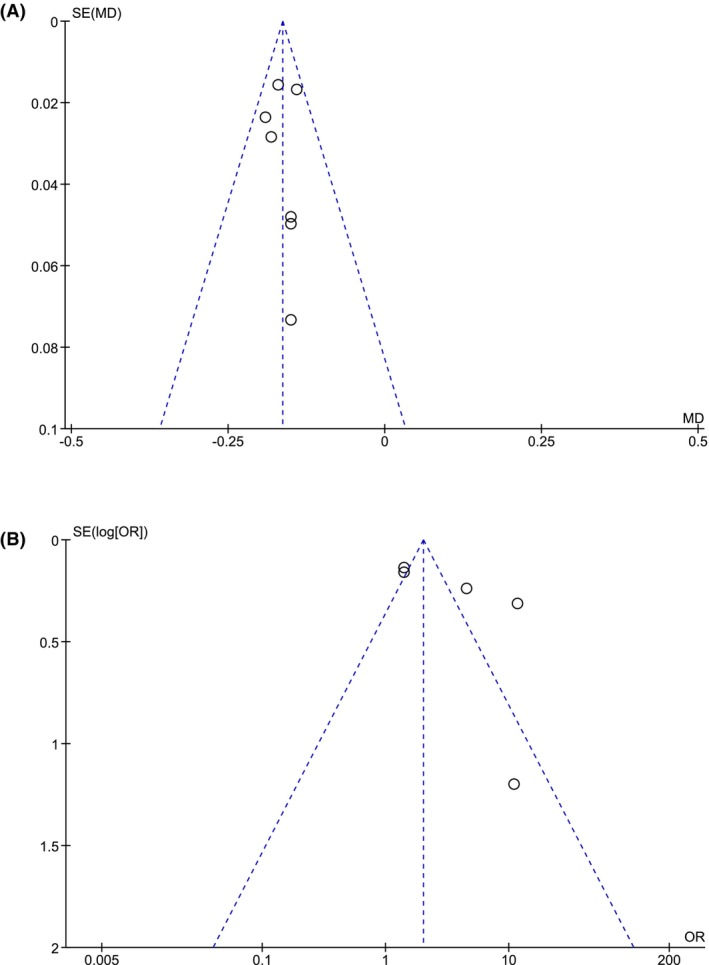
Funnel‐plot analysis (A) LACV in the anterior wall of patients with AF recurrence versus those without [continuous variable] (B) Slow LACV in the anterior wall and AF recurrence [adjusted odds ratio]. AF, atrial fibrillation; LAC, left atrial conduction velocity.

Funnel‐plot analysis showed symmetrical funnel plot for the mean difference (Figure 5A), but slightly asymmetrical funnel plot for adjusted odds ratio (Figure 5B). Egger's test showed no significant small study effects for the mean difference and adjusted OR (*p* > .05).

## 4 DISCUSSION

This meta‐analysis found that patients with AF recurrence has slower LACV in the anterior wall and slow LACV (0.70–0.88 m/s) in the anterior wall was associated with increased AF with AUROC of 0.80 sensitivity of 70%, and specificity of 76%. A cut‐off of 0.80–0.88 m/s for risk stratification is reasonable. Among the included studies, additional ablation beyond PVI does not lower the rate of AF recurrence in patients with slow LACV. Leave‐one‐out sensitivity analyses confirmed the robustness of analysis.

Understanding the cause‐effect relationship of slow LACV with atrial fibrillation (AF) in a broader context is crucial. Tissue fibrosis occurs due to various insults, leading to intercellular gap junction remodeling and abnormal distribution, which results in altered conduction and atrial strain.^13^, ^14^, ^15^, ^16^ Myocardial fibrosis promotes remodeling, which increases the resting membrane potential of myocytes, causing decreased conduction velocity and resulting in shortened action potential duration and refractory period. These conduction disturbances may contribute to the development of re‐entrant circuits in AF. Hence, slow LACV may serve as an indicator of left atrial remodeling or fibrosis and is closely related to the development and recurrence of AF.^9^ Changes in left atrial diameter and left atrial volume index are well‐known indicators of atrial remodeling. However, these measures are limited in reflecting the extent of atrial substrate alteration due to atrial fibrosis.^17^, ^18^, ^19^ Low voltage areas and slow conduction velocities more accurately depict atrial fibrosis and interatrial conduction block, which are closely associated with conduction issues and an increased risk of AF recurrence.^10^, ^16^ Ohguchi et al. demonstrate that anterior LACV was an independent predictor of AF recurrence, but not posterior LACV model which was not significant in univariate analysis.^8^ Okubo et al. also demonstrate that LACV in the anterior wall was independently associated with AF recurrence, but not posterior wall and left atrial appendage.^9^ It has been shown that the mean voltage was significantly lower in the anterior wall compared to the posterior wall in patients with AF and the conduction velocity of the anterior wall might be more sensitive to atrial tissue damage, thus making LACV of the anterior wall as the most important indicator among the other pathway.^20^, ^21^, ^22^ Thus, the current evidence suggested that slow LACV in the anterior wall to be the most important indicator, compared to LACV in the other region of left atrium.

Determining the association between slow LACV and AF recurrence may pave the way for utilizing high‐density mapping systems and noninvasive cardiac studies to detect slow conduction by identifying prolonged P‐wave duration or regions of potential re‐entry, such as left atrial heterogeneity through late gadolinium enhancement (LGE).^23^ This may improve the prognostication of AF recurrence post‐PVI. Identifying LACV as an independent risk factor for recurrence enhances our understanding of the pathophysiology of conduction disturbances in AF and encourages further studies to elucidate the benefits of post‐PVI ablation and the development of new ablation strategies.

### 4.1 Clinical and research implications

While catheter ablation for paroxysmal AF yields satisfying outcomes in terms of AF recurrence, persistent AF remains associated with high recurrence rates. Therefore, patients with persistent AF may require different ablation strategies. However, studies on additional ablation techniques beyond PVI have generally shown disappointing results. This study demonstrates that slow LACV in the anterior wall can be used to stratify patients at risk for AF recurrence. Additionally, slow LACV in the anterior wall can identify potentially relevant re‐entry circuits, which may serve as ablation targets. Nevertheless, whether ablation strategies incorporating substrate modification based on slow LACV in the anterior wall will lead to improved outcomes remains uncertain. Considering the currently available evidence, the authors suggest ablating areas with slow LACV in patients with recurrent AF. Future research is needed to address this evidence gap regarding additional ablation strategies based on slow LACV findings in the anterior wall.

### 4.2 Strength and limitations

The strength of this meta‐analysis lies in its being the first study to pool data on ROC, sensitivity, and specificity, which individual studies cannot evaluate, thereby providing quantitative insights into their importance and reliability. Additionally, there is currently no meta‐analysis that aggregates data on the importance of LACV in the anterior wall. Therefore, this study serves as the first meta‐analysis to highlight the significance of slow LACV in the anterior wall. The pooled evidence from this study also serves as a platform for further research on whether ablation strategies beyond PVI could improve outcomes for patients with slow left atrial conduction velocity.

A limitation of this meta‐analysis is that the included studies used different cut‐off points for slow LACV, although the values were generally close to each other. Finding studies with identical cut‐off points is challenging unless an established standard exists. Kurata (2020) reported on patients from March 2017 to November 2018 who did not undergo additional ablation, while Kurata (2022) included patients from August 2017 to August 2019, with and without additional ablation. Since these studies were conducted at the same institute, there might be an overlap in patient populations. Statistically a leave‐one‐out sensitivity analysis indicated that the statistically significant difference remained even after the removal of either study. Additionally, the small number of studies precluded meta‐regression analysis of potential factors influencing the findings. The current body of literature is insufficient to determine the best ablation strategies for patients with slow LACV in the anterior wall, necessitating further research.

## 5 CONCLUSION

Slow LACV in the anterior wall was associated with AF recurrence after catheter ablation.

## AUTHOR CONTRIBUTIONS

AAL: Conceptualization, Data Curation, Investigation, Formal Analysis, Writing—Original Draft, Writing—Review and Editing. WMR: Data Curation, Investigation, Formal Analysis, Writing—Original Draft. RP: Conceptualization, Data Curation, Investigation, Formal Analysis, Methodology, Supervision, Writing—Original Draft, Writing—Review and Editing.

## FUNDING INFORMATION

None.

## CONFLICT OF INTEREST STATEMENT

Authors declare no conflict of interests for this article.

## ETHICS STATEMENT

Not Applicable.

## References

[1] Pranata R , Karwiky G , Iqbal M . Very‐high‐power short‐duration ablation versus conventional ablation for pulmonary vein isolation in atrial fibrillation: systematic review and meta‐analysis. Arrhythmia Electrophysiol Rev. 2023;12:12. 10.15420/aer.2023.19 PMC10762667 38173799

[2] Dixit S , Marchlinski FE , Lin D , Callans DJ , Bala R , Riley MP , et al. Randomized ablation strategies for the treatment of persistent atrial fibrillation RASTA study. Circ Arrhythm Electrophysiol. 2012;5(2):287–294. 10.1161/CIRCEP.111.966226 22139886

[3] Verma A , Jiang C , Betts TR , Chen J , Deisenhofer I , Mantovan R , et al. Approaches to catheter ablation for persistent atrial fibrillation. N Engl J Med. 2015;372(19):1812–1822. 10.1056/NEJMoa1408288 25946280

[4] Pranata R , Vania R , Huang I . Ablation‐index guided versus conventional contact‐force guided ablation in pulmonary vein isolation—systematic review and meta‐analysis. Indian Pacing Electrophysiol J. 2019;19(4):155–160. 10.1016/j.ipej.2019.05.001 31132409 PMC6697487

[5] Pranata R , Yonas E , Vania R . Prolonged P‐wave duration in sinus rhythm pre‐ablation is associated with atrial fibrillation recurrence after pulmonary vein isolation—a systematic review and meta‐analysis. Ann Noninvasive Electrocardiol. 2019;24(5):e12653. 10.1111/anec.12653 30983090 PMC6931719

[6] Kurata N , Masuda M , Kanda T , Asai M , Iida O , Okamoto S , et al. Slow whole left atrial conduction velocity after pulmonary vein isolation predicts atrial fibrillation recurrence. J Cardiovasc Electrophysiol. 2020;31(8):1942–1949. 10.1111/jce.14582 32445427

[7] Kurata N , Masuda M , Kanda T , Asai M , Iida O , Okamoto S , et al. Left atrial localized low‐voltage areas indicate whole left atrial electrophysiological degeneration in atrial fibrillation patients. Circ J. 2022;86(2):192–199. 10.1253/circj.CJ-21-0527 34707070

[8] Ohguchi S , Inden Y , Yanagisawa S , Fujita R , Yasuda K , Katagiri K , et al. Regional left atrial conduction velocity in the anterior wall is associated with clinical recurrence of atrial fibrillation after catheter ablation: efficacy in combination with the ipsilateral low voltage area. BMC Cardiovasc Disord. 2022;22(1):1–11. 10.1186/s12872-022-02881-6 36319975 PMC9628089

[9] Okubo Y , Oguri N , Sakai T , Uotani Y , Furutani M , Miyamoto S , et al. Conduction velocity mapping in atrial fibrillation using omnipolar technology. Pacing Clin Electrophysiol. 2024;47(1):19–27. 10.1111/pace.14899 38041418

[10] Qi D , Guan X , Liu X , Liu L , Liu Z , Zhang J . Slow conduction velocity predicts atrial fibrillation recurrence after radiofrequency ablation. J Cardiovasc Electrophysiol. 2024;35(3):461–468. 10.1111/jce.16193 38282308

[11] Sato T , Fukaya H , Oikawa J , Saito D , Matsuura G , Arakawa Y , et al. Reduced atrial conduction velocity is associated with the recurrence of atrial fibrillation after catheter ablation. Heart Vessels. 2022;37(4):628–637. 10.1007/s00380-021-01952-6 34613425

[12] Gu Y , Wang H , Xue G , Guo Y , Wu P , He J , et al. Left atrial electrophysiological properties after pulmonary vein isolation predict the recurrence of atrial fibrillation: a cohort study. Rev Cardiovasc Med. 2024;25(5):167. 10.31083/j.rcm2505167 39076500 PMC11267206

[13] McDowell KS , Vadakkumpadan F , Blake R , Blauer J , Plank G , Macleod RS , et al. Mechanistic inquiry into the role of tissue remodeling in fibrotic lesions in human atrial fibrillation. Biophys J. 2013;104(12):2764–2773. 10.1016/j.bpj.2013.05.025 23790385 PMC3686346

[14] Xie Y , Garfinkel A , Camelliti P , Kohl P , Weiss JN , Qu Z . Effects of fibroblast‐myocyte coupling on cardiac conduction and vulnerability to reentry: a computational study. Heart Rhythm. 2009;6(11):1641–1649. 10.1016/j.hrthm.2009.08.003 19879544 PMC3013501

[15] Mahnkopf C , Badger TJ , Burgon NS , Daccarett M , Haslam TS , Badger CT , et al. Evaluation of the left atrial substrate in patients with lone atrial fibrillation using delayed‐enhanced MRI: implications for disease progression and response to catheter ablation. Heart Rhythm. 2010;7(10):1475–1481. 10.1016/j.hrthm.2010.06.030 20601148 PMC3106345

[16] Van Campenhout MJH , Yaksh A , Kik C , Jaegere PP , Ho SY , Allessie MA , et al. Bachmann's bundle a key player in the development of atrial fibrillation? Circ Arrhythm Electrophysiol. 2013;6(5):1041–1046. 10.1161/CIRCEP.113.000758 24129206

[17] Njoku A , Kannabhiran M , Arora R , Reddy P , Gopinathannair R , Lakkireddy D , et al. Left atrial volume predicts atrial fibrillation recurrence after radiofrequency ablation: a meta‐analysis. Europace. 2018;20(1):33–42. 10.1093/europace/eux013 28444307

[18] Soga F , Tanaka H , Mochizuki Y , Mukai J , Suto M , Takada H , et al. Combined assessment of left atrial volume parameters for predicting recurrence of atrial fibrillation following pulmonary vein isolation in patients with paroxysmal atrial fibrillation. Echocardiography. 2019;36(5):862–869. 10.1111/echo.14315 30908731

[19] Liao YC , Liao JN , Lo LW , Lin YJ, Chang SL, Hu YF, et al. Left atrial size and left ventricular end‐systolic dimension predict the progression of paroxysmal atrial fibrillation after catheter ablation. J Cardiovasc Electrophysiol. 2017;28(1):23–30. 10.1111/jce.13115 27779351

[20] Kogawa R , Okumura Y , Watanabe I , Nagashima K , Takahashi K , Iso K , et al. Left atrial remodeling: regional differences between paroxysmal and persistent atrial fibrillation. J Arrhythmia. 2017;33(5):483–487. 10.1016/j.joa.2017.06.001 PMC5634672 29021854

[21] Katritsis D , Sougiannis D , Giazitzoglou E , Kourlaba G , Ellenbogen KA . Regional endocardial left atrial voltage and electrogram fractionation in patients with atrial fibrillation. J Cardiovasc Electrophysiol. 2008;19(12):1254–1258. 10.1111/j.1540-8167.2008.01265.x 18665874

[22] Huo Y , Gaspar T , Pohl M , Sitzy J , Richter U , Neudeck S , et al. Prevalence and predictors of low voltage zones in the left atrium in patients with atrial fibrillation. Europace. 2018;20(6):956–962. 10.1093/europace/eux082 28605524

[23] Fukumoto K , Habibi M , Ipek EG , Zahid S , Khurram IM , Zimmerman SL , et al. Association of Left Atrial Local Conduction Velocity with late gadolinium enhancement on cardiac magnetic resonance in patients with atrial fibrillation. Circ Arrhythm Electrophysiol. 2016;9(3):e002897. 10.1161/CIRCEP.115.002897 26917814 PMC4772170

